# Exploring the Interplay of Social and Physical Factors in Risk Dynamics and Transitions Across the Life-Course of Female Sex Workers in Blantyre, Malawi: A Longitudinal Narrative Study

**DOI:** 10.1007/s10461-025-04790-z

**Published:** 2025-06-27

**Authors:** Wezzie Stephanie Lora, Doreen Sakala, Amr Saidi, Wezzie Nyapigoti, Esnart Sanudi, Maryam Shahmanesh, Frances M. Cowan, Joanna Busza, Nicola Desmond

**Affiliations:** 1 Department of Public Health, Malawi Liverpool Wellcome Programme, Chipatala Road, Queen Elizabeth Central Hospital Campus, Blantyre, Malawi; 2 Department of International Public Health, Liverpool School of Tropical Medicine, Liverpool, UK; 3 Africa Health Research Institute, Durban, South Africa; 4 University College London, London, UK; 5 Key Populations Department, Center for Sexual Health and HIV/AIDS Research, Harare, Zimbabwe; 6 London School of Hygiene and Tropical Medicine, London, UK

**Keywords:** Sex work, Female sex workers, Sex work transitions, Sexual risk, HIV, Malawi

## Abstract

Sexual risk amongst female sex workers (FSW) varies across the life-course and is influenced by socio-economic and interpersonal factors that affect behavioural choices and engagement in HIV/STI care. We explored transitions in the life-course of FSW to understand the dynamics of sexual risk in Blantyre, Malawi. We implemented a nested longitudinal qualitative study as part of the AMETHIST Consortium, a study testing approaches to reduce HIV transmission in sex work. We conducted consecutive narrative interviews with 30 FSW at three-time points over 12 months, with a three- to four-month break between each time point. We compared narratives to understand sex work transitions, HIV risk and engagement with HIV services. We identified factors (social and physical) related to sexual risk at the points of (1) transitions into sex work, (2) continuing sex work, and (3) breaks in sex work. At the entry stage, sexual risk was heightened when women lacked the knowledge and skills for protection against HIV/STI. Whilst continuing sex work, women’s immediate financial needs were prioritised over their HIV/STI risk. These behaviours occurred whether they were aware of the associated HIV/STI risk. During breaks, women perceived lower risk and reduced engagement in prevention strategies, particularly when they had stable partners, which paradoxically increased their risk. These narratives reveal how social context informs and limits access to health care while concurrently promoting risky behaviours. A multifaceted and dynamically responsive approach that considers risk differentiation from a temporal perspective can strengthen targeted interventions, effectively addressing the multiple challenges faced by FSW.

## Introduction

Sex work is an evolving process marked by various transitions across the life-course, and each transition presents risks for female sex workers (FSW). Sex work in Southern Africa, as elsewhere, is associated with a high risk of HIV infection, driven by both physical and social factors [1]. Physically, FSW are exposed to HIV acquisition from inconsistent condom use, multiple sexual partners, and limited control over sexual interactions, which are also associated with poor mental health and substance use [2, 3]. Socially, they may experience stigma, discrimination, and violence, which further exacerbate their vulnerability to HIV and restrict their access to healthcare services [1, 4, 5]. These factors interact in complex and dynamic ways, likely fluctuating over their life-course.

Despite the widespread availability of prevention strategies, FSW continue to bear a disproportionate burden of HIV infection in Southern Africa. One key challenge is the failure of interventions to adequately identify and target al.l individuals engaged in sex work, including those who may not self-identify as FSW and new entrants [5, 6]. These populations include individuals who engage in occasional or transactional sex but do not consider themselves FSW, as well as those operating in informal or clandestine settings. These women may remain hidden or “invisible” to services. Additionally, limited data on the size and location of FSW populations impede efforts to design and implement targeted HIV prevention and care services [6, 7].

Existing programmes often find young women and new entrants into sex work particularly hard to reach, missing crucial opportunities for early intervention and support [8]. Initiatives like Determined, Resilient, Empowered, AIDS-free, Mentored and Safe (DREAMS) that target young women with vulnerabilities known to be associated with selling sex face challenges in reaching this population [9–12]. As a result, these individuals often lack access to comprehensive sexual and reproductive health services, including contraception and HIV prevention methods, placing them at increased risk of unintended pregnancy and HIV/STI infection. Failing to reach young women and new entrants in sex work programmes represents a significant gap in HIV prevention and care. Continued efforts are needed to integrate these aspects more effectively for early intervention and effective harm reduction [13].

While the existing body of research acknowledges the broader challenges faced by FSW, there is limited knowledge about the relationship between specific drivers influencing sexual risk behaviours and the transformative moments FSW experience throughout their sex work journey in Southern Africa. These transitions can include entering sex work, moving between different venues or types of sex work, and ageing within the profession [14–16]. Each stage brings unique physical and social risks. For instance, younger FSW may face higher levels of exploitation and violence, while older FSW may struggle with declining health and reduced client demand [17].

In Malawi, FSW have a high burden of HIV (49.9%) despite the availability of prevention, treatment, and care [18]. This vulnerability is compounded by early life adversities, cumulative disadvantage, and the inherent risks associated with sex work. This paper explores how social drivers inform transitions and how transitional moments produce heightened risk exposure, thereby informing more targeted and effective interventions that acknowledge that involvement in selling sex remains a dynamic rather than a static experience.

## Methods

### Study Context

This study was part of the multi-country Adapted Microplanning: Eliminating Transmissable HIV in Sex Transactions (AMETHIST) Consortium. We explored whether risk-differentiated microplanning with self-help groups amongst FSW would reduce the risk of HIV acquisition and transmission in Zimbabwe, with formative work in Malawi and South Africa to identify the potential transferability of trial findings to other Southern African contexts [19]. Research across the three countries focused on understanding the heterogeneity of sex work experiences and service engagement amongst FSW, specifically how risk perceptions, risk behaviour, and engagement with services changed over time.

In Malawi, the AMETHIST was conducted in Blantyre in the southern region. This location is strategically chosen because it has one of the highest HIV prevalence amongst the general populations in Malawi (11.9%) [20]. There were 3600 FSW with an estimated HIV prevalence of 57% [21]. Data collection was carried out between February 2022 and January 2023.

### Study Design

As part of a mixed methods design for AMETHIST Malawi, we conducted a longitudinal ‘life-course’ study using narrative interviews with 30 FSW recruited from a bio-behavioural survey undertaken at the same time. Narrative interviews were conducted at three-time points over 12 months, with a three- to four-month break between each. In this paper, we focus on the data from these narrative interviews.

### Participants and Life-Course Narrative Interviews

We defined FSW as those living or working (for at least one month) in urban Blantyre and who sold sex (exchanged sex for money or goods in the past 30 days) during the recruitment period (February-March 2022). Participants were purposively sampled to reflect a range of ages, sex work typologies (street-, home- and venue-based), and duration of sex work (Table 1). Questions about where FSW solicited clients, duration of sex work and current sex work prices in the geographical area were used to determine the level of women’s involvement in sex work.

**Table 1:** Participants’ socio-demographic characteristics, *N* = 30

No.	People	Age (Avg 32.8 Med 33 Range 21–57)	Age of sex work entry (Avg 25.34 Med 24.5 Range 15–44)	Years in sex work (Avg 7.34 Med 6 Range − 1–19)	Marital status (Divorced 66.67% Never married 26.67% Widowed 6.67%)	Education level (Secondary 73.33% Primary 26.67%)	Programme engagement (Yes 70% No 30%)
1	Isabella	36	15	15	Divorced	Secondary	Yes
2	Thoko	32	27	5	Divorced	Secondary	Yes
3	Annabel	34	17	13	Never married	Secondary	Yes
4	Olive	37	18	19	Divorced	Secondary	Yes
5	Sahara	36	17	19	Never married	Secondary	Yes
6	Jane	40	33	8	Divorced	Secondary	Yes
7	Kele	23	18	6	Never married	Secondary	Yes
8	Acacia	33	25	3	Never married	Secondary	Yes
9	Silvia	40	37	3	Divorced	Secondary	Yes
10	Yamie	33	31	3	Divorced	Primary	No
11	Tricia	27	22	6	Divorced	Primary	No
12	Shakira	38	30	6	Divorced	Primary	No
13	Magda	30	18	13	Never married	Secondary	Yes
14	Clara	30	23	8	Never married	Secondary	Yes
15	Natasha	27	24	4	Divorced	Secondary	Yes
16	Martha	43	36	8	Divorced	Primary	Yes
17	Lufi	21	16	6	Never married	Secondary	Yes
18	Savannah	35	29	7	Divorced	Primary	Yes
19	Bertha	57	44	14	Widowed	Secondary	Yes
20	Lucia	24	23	-1	Never married	Secondary	No
21	Carol	28	27	2	Divorced	Secondary	No
22	Charo	24	23	1	Divorced	Secondary	Yes
23	Chipie	32	28	5	Divorced	Secondary	No
24	Petra	29	22	8	Divorced	Secondary	No
25	Cathy	27	19	9	Divorced	Primary	Yes
26	Cynthia	35	30	6	Divorced	Primary	Yes
27	Titha	36	27	10	Widowed	Secondary	Yes
28	Chimze	34	27	8	Divorced	Secondary	Yes
29	Agatha	38	34	2	Divorced	Secondary	No
30	Penny	25	21	5	Divorced	Primary	No

All names presented in the table are pseudonyms

We conducted life-course narrative interviews to understand individuals’ lives within the context of their socio-cultural and historical environment over time [22]. Life-course narratives explore how early life experiences shape later outcomes and how historical events impact individual trajectories. In sex work research, life-course narrative studies help unravel the complexities of FSW’s experiences, transitions and vulnerabilities over time. By exploring the trajectories of individuals engaged in sex work, researchers can identify critical junctures where risks are heightened to inform targeted risk mitigation interventions [15]. A team of four trained qualitative researchers (both male and female) with extensive experience working with high-risk populations conducted all interviews. Previous studies in similar contexts have shown that experienced and trained male researchers can effectively conduct research amongst FSW without posing significant challenges [23, 24] dependent on the quality of rapport and trust built between the researcher and the participant.

**Fig. 1:**
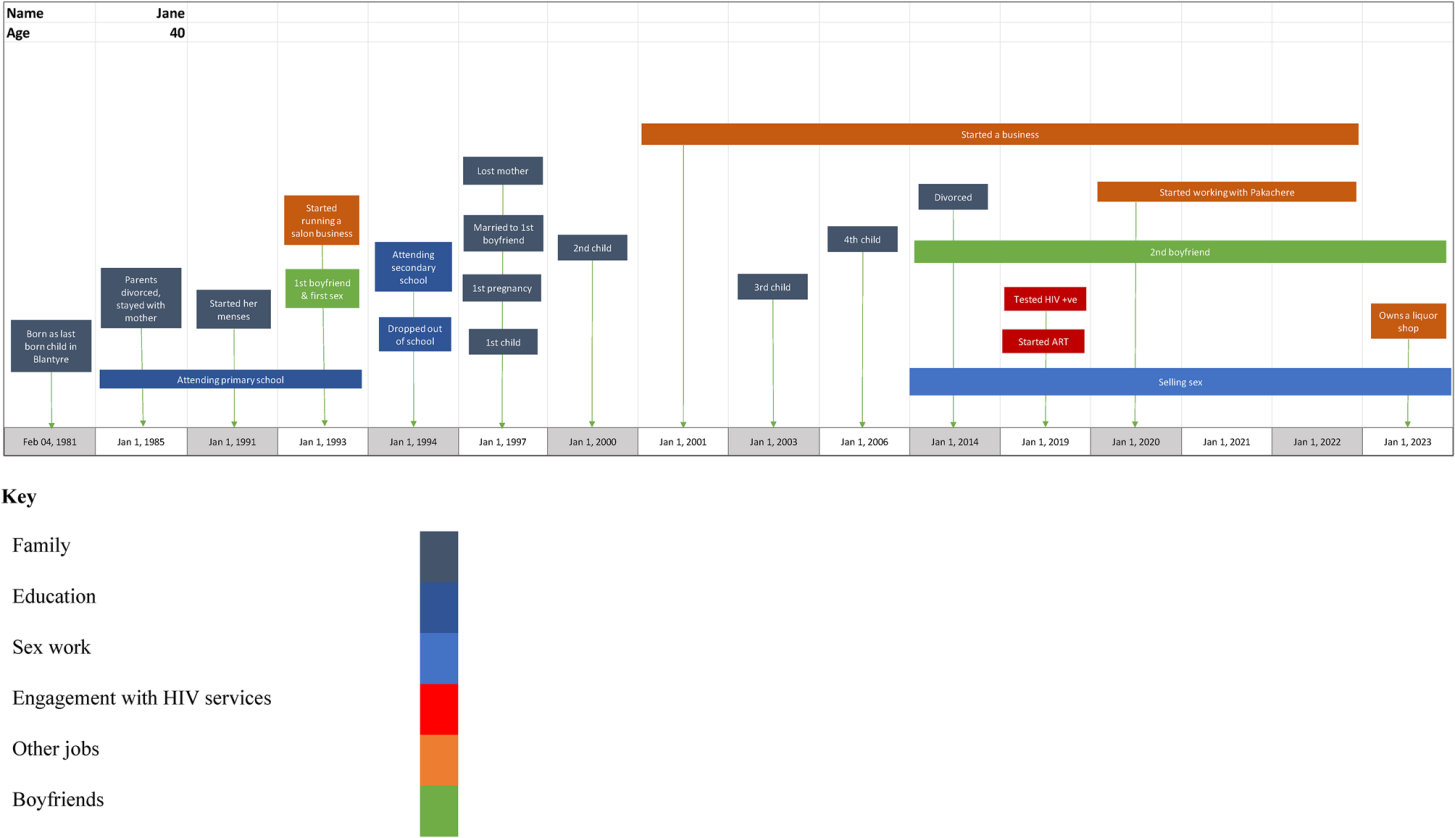
An example of a life-course narrative grid

The research team visited potential participants in their homes or at venues, and the informed consent process preceded full enrolment and rolling recruitment to the study. Following consent, FSW were invited to participate in three consecutive narrative interviews. Interviewers guided women to talk about their transitions into sex work, risky behaviours and perceptions, experiences with HIV care and prevention, and engagement with targeted sex work interventions at different phases of their lives. We also explored the environments in which women who sold sex lived and worked, as well as their strategies for risk mitigation and resilience. A life grid (see Fig. 1 for an example) was co-developed with each participant during their first interview and used to guide subsequent discussions during follow-up interviews, clarifying transitions and underlying influences [24]. Participants were followed-up for two more rounds of interviews. During follow-up interviews, we validated the data collected in the previous interview, assessed how and how often women transitioned into and out of sex work and how these transitions affected their risk perceptions, behaviour, exposure and encounters with all types of health services. We were interested in understanding how often transitions occur, at what age and stage in the sex work life-course, and how these shaped FSW’s perceptions and responses to health-related risk. The life grids were designed to be two-fold: (1) to understand retrospective experiences since women began selling sex and early life experiences that contributed to this and (2) to prospectively explore changes in their lives over one year from recruitment in the study. The life grids were updated at each follow-up interview. All interviews were conducted in Chichewa (the local language) and were responsive to individual priorities, both in terms of issues around confidentiality and in the direction of the narrative itself.

### Data Management and Analysis

Qualitative data were collected through digital audio recorders. Life grids were manually produced on paper during the initial interview, revisited and refined on paper at each subsequent visit, and reproduced through Excel. Audio files were transcribed verbatim and translated into English. All files were anonymised and saved on password-protected computers. All data were transferred to NVivo 12 (QSR, Melbourne, Australia) for data management and to support analysis.

Thematic analysis [25] combined with framework analysis was conducted to identify physical and social drivers of sexual risk and healthcare-seeking inductively and deductively amongst FSW. First, we familiarised ourselves with the data by reading and re-reading transcripts, noting key patterns in participants’ narratives. The research team then manually coded a preliminary set of eight transcripts independently, and discrepancies were resolved through consensus discussion with the lead social scientists (WSL and ND). A codebook was developed once the research team was satisfied that the coding was consistent across individual coders. The remaining transcripts were single-coded in NVivo, with WSL reviewing at least every fifth document. These codes were clustered into broader themes. The team reviewed and discussed the emerging themes regularly, which were iteratively adapted as coding progressed. We used framework analysis [26] to systematically map participants’ life grids into a structured framework, highlighting key transitions and major life events such as age at entry into sex work, pregnancies, relocations, deaths, and service engagement. This approach allowed us to analyse the entire dataset within a single framework, facilitating a comparative view of participants’ experiences. Once all data had been coded and the framework developed, the full team met for a data analysis workshop. During this, connections between sexual risk and transitions were made to identify patterns. We compared behaviour or experience in key areas across all participants, which provided a more nuanced understanding of sex work transitions, periods of risk, and engagement with care with a focus on capturing a diversity of experiences. Themes were refined through iterative review to ensure coherence and validity. Finally, we defined and named themes to capture the complexities of transitioning into sex work, integrating them into the analysis with participant quotes [26].

## Results

We framed risk at two levels: (1) Physical drivers of risk were characterised as factors that would directly reduce or exacerbate the risk of HIV infection; these were Pre-Exposure Prophylaxis (PrEP)/Post-Exposure Prophylaxis (PEP)/Antiretroviral Therapy (ART) engagement, condom use, number of partners and type of sex; (2) Social drivers were those that might indirectly affect women’s risk of HIV infection, these were alcohol and substance use, gender-based violence (GBV), age, type of partner, socialisation, family structures, income, relationship status, education, employment, responsibilities, modern lifestyles, social events, seasons, and relocation/migration.

We identified factors (social and physical) related to sexual risk at the points of (1) transitions into sex work, (2) continuing sex work, and (3) breaks in sex work (temporary). We did not interview women who had permanently transitioned out of sex work due to the nature of the recruitment from a survey of women who currently define themselves as FSW.

We present the findings by stage within sex work life-courses, highlighting the intersectional influences of physical and social drivers at each stage and how these drivers impact risk exposures during transitional moments in FSW’s lives (Table 2).

**Table 2:** Key HIV risks at transition points and implications for interventions

Transition point	HIV risk	Intervention implication
Transition into sex work The liminal stage	**Physical drivers** Condomless sex High number of partners (new FSW) Limited HIV service engagement (HIV testing, PEP & PrEP)	Peer-led outreach programmes targeting new entrants to sex work. Comprehensive orientation packages including HIV prevention tools and, stigma and harm reduction strategies GBV response services integrated with HIV programming
	**Social drivers** Young age Alcohol and substance use Stigma GBV	
Continuing sex work Slowing down in sex work	**Physical drivers** Sporadic engagement with ART/PrEP Decreased condom negotiation power due to stable regular/permanent partners relationships	Differentiated service delivery models to accommodate changing schedules Economic empowerment programmes to reduce financial vulnerability GBV response services integrated with HIV programming Stigma reduction strategies
	**Social drivers** Alcohol and substance use Persistent stigma GBV Economic pressure due to fewer clients	
Temporary transition out of sex work Breaks/pauses in sex work Re-entry into sex work	**Physical drivers** Sporadic engagement with ART/PrEP, Normalisation of condomless sex with stable regular/permanent partners, STI prevalence Challenges with re-engagement into care after a break	GBV response services integrated with HIV programming Peer bridging programmes maintaining contact during breaks from sex work Re-entry support packages including HIV testing and PrEP restart Economic empowerment programmes to reduce financial vulnerability Stigma reduction strategies
	**Social drivers**: GBV, Alcohol and substance abuse, Intensifies stigma during transition periods Economic pressure due to unstable income	

### Transitioning into Sex Work

Transitioning into sex work refers to the time when a woman begins to exchange sex for money or gifts, regularly or irregularly and either formally or informally. Narratives surrounding entry into sex work reveal diverse pathways that are shaped by differing degrees of agency, social support and economic necessity influenced by familial background and preceding, often catastrophic life experiences. During this time, changes in sexual risk were shaped by the gradual process of sex work entry, usually experienced before introduction into sex work and sustained in sex work. These variations in entry routes have implications for how women engage with HIV/STI services.

In the first pathway, the woman’s entry into sex work is depicted as a conscious and pragmatic choice driven by the need to secure an income in the context of household poverty.

*“I dropped out of school because there was no money for school fees*,* so I started searching for piecework*.

*whilst in the village*,* but there was none*,* and there was no money to start a business*,* so I decided to come*.

*here and start sex work.”* **(Silvia**,** 40 years old)**.

The second pathway emphasises the role of circumstantial pressure in the transition process as a response to the overwhelming responsibilities of childcare following her divorce. This route suggests a constrained choice, where external pressures limit the woman’s control over her circumstances.

*“I was confused after the divorce; I didn’t have any money and didn’t know how I was*.

*going to raise the children so I started sex work.”* **(Thoko**,** 32 years old)**.

In contrast, the third pathway portrays a woman who enters sex work through an active and self-affirmation decision. Her choice to engage in sex work is validated through the support she receives from her social network.

*“When my charcoal business failed to thrive*,* I expressed my frustration with poverty to my mother and that I had decided to pursue sex work as a means of income. Realising the potential of using my body to earn money*,* I made the difficult choice to enter sex work after seeking advice from friends.”* **(Penny**,** 28 years old)**.

Actual introduction to sex work was often a gradual process. All participants reported that they were introduced to sex work by either a friend, relative or acquaintance; none made the decision alone. Yamie’s acquaintance from her community, who was familiar with the challenges she faced, recommended sex work as a solution, although Yamie had been a committed churchgoer and initially hesitated to start sex work:

*“I met a certain friend*,* she said sister*,* you might like it or hate it; because at that time I was prayerful and when I say prayerful you should understand. So*,* I refused when the person told me for the first time. She went and when she came back*,* I told her to pick me up on her next visit…we went there [to sell sex] and saw that as the sun rose*,* I would have some money to send the children to buy food. When I had a lot more*,* I would say buy some maise and keep it. That was how I started.”* **(Yamie**,** 34 years old)**.

Gradual entry into sex work was also underpinned by more deceitful and persuasive tactics of peer relations, such as Thoko, who was deceived into sex work through a friend who promised her an employment opportunity:

*“My friend persuaded me to go with her to Mozambique with the belief that she’s found me a job as a maid*,* this was all a lie*,* there was no job for me. She brought me to a bar and asked me to wait for some time as she sorted out issues with my post. I had no money for food or transportation to go back to Malawi*,* eventually*,* she told me that paid sex was the only option for me to sustain my livelihood in the foreign land.”* **(Thoko**,** 32 years old)**.

Thoko’s example suggests that forms of ‘trafficking’ within peer networks exist in this context. The promise of non-existent employment was also common amongst women relocating from rural to urban areas within Malawi.

At this stage, those new to sex work reported more risky behaviours. Taking marijuana and drinking beer were reported as *“…a coping mechanism and fitting in this unfamiliar environment”* **(Annabel**,** 30 years old)** amongst those who reported having never done so before joining sex work. For many, this was very likely a way to cope with the stigma and depression likely to accompany this phase of sex work when women are adjusting to their new social identity. Lufi’s account highlights the profound psychological challenges that can arise through engagement in sex work, leading to harmful behaviours such as abuse and suicidal ideation.

*“I started drinking beer not too long ago. It began because I was struggling with a lot of thoughts – you know in this job a lot happens*,* I was wishing I could be with my mother*,* sometimes thinking about suicide. Everything I was going through weighed heavily on me”* **(Lufi**,** 21 years old)**.

Some women lacked knowledge and information about sexual reproductive health services available in the communities, including HIV services. However, sometimes peers could help to mitigate risk by teaching newcomers how to protect themselves and use condoms, as described by Annabel:

*“Since I was new*,* she taught me how to put a condom on a man. She used to tell me that this is how much you charge customers for sex with or without a condom.”* **(Annabel**,** 34 years old)**.

At this stage, sexual risk was commonly heightened when women were neither fully integrated into sex work nor entirely outside of it. During this stage, women are not fully equipped with the knowledge and skills to protect themselves against HIV/STI. The gradual entry involved experimentation due to the novelty of engaging in sexual activity for the first time or with a new partner, and this novelty had the potential to increase risk-taking behaviours as women navigated unfamiliar territory. Once women had entered this social space, they described experiencing mixed emotions, including excitement, intimidation, and fear. This commonly highlighted the transition to a new culture where women assimilated new norms, values, risks, benefits, and boundaries. The need to rely on peers was thus increased to assist them in orientation and support. What one was told about sex work, when and where to access clients, and when and where to access health services was important information for newcomers to the industry as it set them on a path to determine how best they could minimise risk in navigating their future life as a sex worker.

### Continuing Sex Work

Life in sex work was characterised by a mix of opportunities, uncertainties, and inevitabilities in negotiating risk within the sex work environment, all of which affected individual risk perceptions. Most women entered sex work to improve livelihoods for themselves and their families, often in response to catastrophic events. Yet after this early stage of vulnerability, many described how sex work provided opportunities and reported positive changes in their lives as a direct result of their work, such as sending their children or dependents to school, building a house in the village and the ability to farm, often independently from a man. Both Charo and Acacia emphasised the benefits and opportunities that sex work had given them:

*“I think sex work is going well because I can pay my child’s school fees*,* daily food and rental bills. My life is okay.”* (**Charo**,** 25 years old)**.

*“It changed my life because I can do things on my own.”* ***(Acacia***,*** 28 years old)***.

However, sex work was also recognised as full of uncertainty and was described by many women as getting lucky. Uncertainties described included whether one could get clients every day or the type of sex the client might request. Prices fluctuated, and clients always negotiated for lower prices. As one could not predict daily earnings and women were reliant on what they earned through sex, they acknowledged their exposure to risks, including unprotected sex, to maximise daily earnings. These were perceived as inevitable and uncontrollable since they were often a consequence of broader socio-political and socio-economic structures. Jane described how her sex work income fluctuated due to the broader socio-economic status in society:

*“It’s not like you can depend on it with the current situation*.^1^*Now you can’t say you will go to a bar and find money to pay rent and bills. That used to happen in the past not currently. In the past it was possible.”* **(Jane**,** 41 years old)**

Perceptions of inevitability were also common in women’s expectations of acquiring HIV, as illustrated by Cynthia below.

*“I found that I was okay*,* but I was still thinking that what I was doing*,* either way*,* I will end up contracting diseases.”* **(Cynthia**,** 35 years old)**.

On the one hand, she feels relatively well at present, suggesting that she engages at some level with health services or prevention methods. On the other hand, she believes that contracting HIV is inevitable, reflecting a pessimistic view of the long-term risks associated with sex work.

At this transitional stage, women’s immediate needs, such as maximising daily earnings, were prioritised over their HIV/STI risk.

Despite extensive awareness of HIV/STI prevention, this inevitability dominated women’s attitudes to their own risk of acquiring HIV since they continued to normalise condomless sex, especially with regular and permanent partners, and multiple partners to increase earnings. Women also reported that they accessed local organisations providing HIV/STI services in their communities and also shared updated information about HIV prevention, treatment, and care, mainly through social networks. This was done either formally (through peer-based intervention strategies driven by local non-governmental organisations (NGOs) or informally through friends. For those who remained reliant on sex work for their primary income for long periods, sexual risks were exacerbated by substance use and alcohol, which decreased their capacity to negotiate safer encounters as well as undermined their health care. Annabel described how she consciously allowed her excessive alcohol consumption to affect her adherence to ART, which she acknowledged was risky:

*“Truthfully*,* I drink alcohol. From Monday to Friday*,* I take the meds on time*,* but on Saturday*,* maybe I get drunk*,* I wake up and see the time is past 10 which means I missed the time and I take them past 10. Sometimes*,* I sleep out with a man and come in the evening*,* which means I do not take the medicine. When I tested the viral load*,* my viral load was high. When they asked me*,* I was honest to say I do not take medicine*,* I take it sometimes*,* sometimes I do not take it*,* and I skip when it is weekends.”* **(Annabel**,** 30 years old)**.

Annabel’s experience was not unique, with poor adherence to HIV treatment largely attributed to forgetting to take pills when intoxicated, further increasing perceptions of the inevitability of falling sick.

### Slowing Down in Sex Work

We defined slowing down in sex work as a reduction in the number of partners and/or working days over time (thus less but not no activity). The main reasons for slowing down included transitions in relationship status, greater financial stability, and a desire to avoid violence following either personal experience (self or peers) or after having heard about violent incidents within their wider social network. Other reasons included getting older and finding sex work more tiring, weather events impacting client numbers, and other reduced sex work demand. Almost all participants had slowed down in sex work at some point in their lives. Agatha describes how her decision was informed by the risk of violence.

*“I slowed down in November and December 2022*,* I was afraid because there were a lot of things happening*,* our friends who were going around could tell us about their experiences like I had been beaten*,* I hadn’t been paid they* [clients] *were just buying beer for me.”* **(Agatha**,** 36 years old)**.

Agatha’s case highlights the emotional toll and safety concerns inherent in sex work that both affected her mental well-being and compelled her to slow down in sex work.

Chimze explains that this was frequently when cold weather made sex work less attractive.

*“I limitedly do that [sex work] because of the cold weather.”* (**Chimze**,** 35 years old**).

Penny’s sex work involved travelling to meet men, and she travelled less when times were more challenging. Penny’s acknowledgement of declining earnings and the strategic adjustment of her work schedule to weekends illustrates the economic instability that many sex workers encounter, but she adapted to maintain her income.

*“It has been a painful life; we are no longer making money as we used to. That’s why we just agreed with my friend that let us just go there during weekends so that we can still make money*,* even though we face stigma*,* but the way it is*,* there are times you meet clients and there are times you don’t meet a client that’s how it is.”* (**Penny**,** 28 years old**).

Isabella explained how growing older translated to preferring familiar men rather than seeking new clients. Isabella’s account highlights personal growth and adaptation over time as someone who was in sex work for a long time. Over time, there was a notable shift in her life-course towards more selective and deliberate client interactions.

*“My life is different to how it was before. In the first days*,* I was able to visit the bar every day but now things have changed. I still go*,* but not every day and the way I was having sex with men has changed as well. Currently*,* I sleep with men whom I knew from way back who happen to phone me*,* or I phone them. In the past*,* I was going to look for men at the bar without knowing who I was going for. I was doing this for almost a whole month. I am now able to visit a bar once a week*,* and I have sex with men well-known to me.”* **(Isabella**,** 36 years old)**.

In no instances were narratives about slowing down linked to a direct desire to reduce HIV risk. When FSW experience periods of reduced activity, income decreases due to fewer clients as such, some women may face economic pressure. This financial stress can lead to accepting higher-risk scenarios they might otherwise decline, agreeing to condomless sex for premium payments when financially desperate or accepting new clients with less thorough screening. As indicated by Isabella FSW might retain longer-term clients who, while potentially lower-risk due to familiarity, might have established patterns of condom non-use.

### Temporary Transitions Out of Sex Work

The cycle of exiting and later returning to sex work after a break was extremely common amongst FSW in this setting. A total of 20/30 participants reported to have transitioned temporarily out of sex work one or more times. Whilst we did not recruit women who used to sell sex but had now stopped entirely, our longitudinal approach meant that four women dropped out of the study over the one-year follow-up period. We traced the reasons for dropping out; two were still in sex work but had relocated outside of Blantyre, and two reported to have gotten married and stopped selling sex.

The narratives highlighted wide variation in the duration of sex work breaks depending on individual circumstances, although this generally ranged from less than one to five years. Despite attempts to seek alternative lifestyles, most women returned to sex work when their circumstances changed for the worse. We identified two types of such transitions.

#### Breaks/pauses in Sex Work

In this context, we refer to sex work breaks/pauses as periods during which women completely ceased engaging in sex work but later re-entered. Relationship status, physical and mental well-being, family obligations and financial stability were common motivations for taking sex work breaks, and FSW described believing some of these to be permanent “exits” at the time.

Sex work breaks were often an active choice for an alternative lifestyle fuelled by the desire for a socially acceptable reputation as a respectable woman in the community. This was generally achieved by establishing a business or finding a husband. Those who reported having a stable partner stated that they had a break because they were someone’s respectable wife or in a serious relationship. Those who ventured into a different business reported that there was no need for them to remain in sex work as they had enough money for their needs.

In other cases, a sex work break was a forced choice because of unforeseen events such as illness, pregnancy, or family obligations. Some women, like Agatha, stated that illnesses like STI made them ‘unattractive’ to clients, or in other cases, their bodies felt so weak that they could not keep up with sex work demands.

*My body becomes weak*,* to the point where you might find it difficult to walk. When you go there whilst sick*,* you won’t get the money.* **(Agatha**,** 36 years old)**

Illness caused by STI made sex work physically uncomfortable, and when the symptoms were obvious to clients, women were prevented from selling sex. If this or other serious illnesses occurred, women often returned to their home village, returning to sex work following recovery.

Silvia got pregnant soon after starting sex work and was forced to stop for a period until the child was old enough to live with her mother:

*I started as a sex worker; I was a sex worker for two months*,* then got pregnant. I wasn’t using any contraception because I never knew what it was to be a sex worker; I was sort of learning on the job.* **(Silvia**,** 40 years old)**

Although some of the women described their work as risky, complex, and stigmatising, 10/30 reported having never taken a break from sex work. This was largely claimed to be due to a lack of financial safety nets and the burden of supporting children, parents and extended families such as Chimze:

*“Laugh! If I take a break*,* what am I going to feed the children? How about food for my parents? If I take a break*,* things will not be good”.* (**Chimze**,** 35 years old**)

Some actively avoided stable partnerships because they did not want to settle down and rely financially on a male partner, whilst others felt lucky that their health had not impacted their ability to work. Women’s narratives revealed that income level and stability could greatly influence FSW’s capacity to take breaks from sex work. Some women felt less at-risk during periods away from sex work, especially when they had found a stable partner who supported them financially. Their engagement in risk prevention strategies such as condom use and accessing targeted services for FSW also decreased during these times, potentially increasing their risk. This paradox was largely due to an unwillingness to be seen or identified as an FSW, prompting them to demonstrate their commitment to one partner through condomless sex. Women often stop taking treatment for HIV or stop using prevention options such as PrEP to demonstrate their changed social status as no longer ‘risky women’. Fear of social stigma related to their previous status was often prioritised over fear of consequences from disengagement with care and failure to continue with prevention options. This disengagement was exacerbated if they had married and relocated elsewhere.

#### Re-entry To Sex Work

Despite aspirations of transitioning permanently out of sex work, women would often re-enter sex work following further changes in personal circumstances. Economic disruption, intimate partner violence, and social marginalisation were key factors described as pushing women who had invested in a new identity back into sex work. Both Chipie and Tricia, for example, re-entered sex work for financial reasons, either following abandonment by a partner or the failure of a business:

*“I stopped when I was with a certain man who was providing me with everything regarding home expenses*,* but he was married so he withdrew*,* and then I started selling sex work again.”* **(Chipie**,** 33 years old)**.

*“I stopped because I had the business but then sometimes*,* I couldn’t support myself*,* so I went back to selling sex”* **(Tricia**,** 29 years old)**.

A few women, particularly those who remained in the same physical locale, described how they struggled to reintegrate into mainstream society and redefine their social identity since people around them knew them as sex workers and continued to see them as such. This limited their choices if they remained within the same communities and pushed them back to sex work.

Following re-entry into sex work, some women grappled with feelings of shame, viewing their return as a failure, which increased levels of self-stigma. These negative emotions hindered women’s ability to re-engage with support services fully. Women would hide from their old peers because they thought they would be laughed at for returning to sex work after leaving with so many hopes for a better future. Annabel described how she felt ashamed and that she was laughed at when she returned following an illness [recurring abdominal pain] episode:

*“The way I have moved around hmm*,* I was embarrassed*,* it was not easy coming back to sex work as women laugh at you.”* **(Annabel**,** 30 years old)**.

Losing touch with previous social networks and programmes that target FSW and shame at having had to come back to sex work after having left led many to delay or avoid re-engagement in HIV prevention services. Savannah highlighted this in her narrative, whilst Tricia’s failure to actively re-engage led to her acquiring HIV.

*When we come back to sex work*,* sometimes we get shy to tell them* [healthcare workers] *what we do. When we leave sex work and come back*,* it is not easy to go back and collect condoms.* **(Savannah**,** 36 years old)**

*The first HIV test was negative; I was only found positive when I came back to sell sex.* **(Tricia**,** 29 years old)**

The emotional discomfort experienced by Savannah and Tricia in re-engaging with services might result in delays in accessing care, leading to missed opportunities for timely intervention and support.

## Discussion

This longitudinal study on FSW in Malawi makes a significant contribution to the existing literature by reconceptualising sex work as a dynamic, non-linear trajectory and mapping HIV risks to the transition points. This fluidity poses challenges for interventions and necessitates a reconsideration of how HIV prevention services are designed and delivered to capture women at multiple life stages, not just during initial vulnerability.

Previous research has predominantly focused on factors influencing either entry into sex work or permanent exit [27–30], creating a dichotomy that fails to capture the complex reality of sex workers’ lived experiences. This binary framing has limited our understanding of the fluid nature of sex work engagement and liminal stages and, consequently, restricted the effectiveness of HIV prevention efforts targeted at this population. Each transition represents a moment of vulnerability to HIV risk, but also an opportunity for targeted intervention. However, in this study women either did not recognise these risks or were aware of them but were constrained to prioritise social and financial pressures over health consequences. This nuanced understanding offers significant implications for HIV prevention science and service delivery.

Slowing down, temporary exits and re-entry into sex work represent significant periods in sex work trajectories that are limited in the current HIV prevention research and interventions. Efforts should be made to conduct longitudinal evaluation research assessing these changes over time to examine these temporal transitions and their outcomes [31]. Our participants experienced gaps in service access during periods of intermittent involvement. These findings align with other studies that FSW have challenges maintaining continuous access to services once initially reached, highlighting a critical gap in the intervention cascade [32]. This oversight leaves a critical gap in our understanding of how to support women during these vulnerable transition periods. This suggests peer interventions, for example, need ‘transition bridging’ components to maintain connections even when women temporarily exit. By understanding these different life stages and associated risks, service providers can offer both spatially and temporally differentiated care responsive to changes in the life-course of women engaged in sex work.

The failed attempts to leave sex work emerged as particularly high-risk periods, often characterised by reduced protective behaviours. Economic necessity, the lack of alternative employment and GBV are widely accepted as key factors driving entry into the sex trade, as well as posing significant barriers to exit [30, 33]. This aligns with findings on the centrality of economic factors in sexual risk amongst women in Swaziland [34]. Current interventions that try to help women exit sex work, such as job training, temporary housing, and counselling produce mixed evidence [35]. This gap in effective exit support affects HIV prevention as women who feel stuck in sex work report using condoms less consistently and get HIV testing less often. Programmes that address both immediate health needs and help women build new livelihoods could reduce HIV risk while supporting women’s choices and well-being. More complete exit interventions that address all the factors that make it hard for women to move into other types of work are needed. Promising approaches might include economic strengthening interventions that target women at key transition points, such as the combination of HIV prevention and microfinance programmes [29, 36]. Such interventions must be flexibly designed to accommodate women’s fluid movements in and out of sex work, rather than requiring complete cessation of sex work to receive support.

We identified GBV, stigma, substance abuse and alcohol dependency as challenges that cut across the life-course of FSW and significantly impact HIV risk management and access to HIV/STI services. Despite their critical importance, services addressing these issues were lacking in this setting. This reflects findings from other studies in similar contexts [37, 38]. Given these findings, there is a clear need for multi-sectoral interventions that prioritise addressing stigma, GBV, substance abuse and alcohol dependency alongside sexual reproductive health.

The focus of the study is significant given the higher prevalence of HIV in Blantyre and amongst FSW, as well as the knowledge gap about sex work over the life-course and differentiated service needs at these various points. The strength of this study draws on its utilisation of a longitudinal life-course narrative approach, allowing for comprehensive mapping of the dynamic life trajectories of FSW to allow new information about transitions/risks to emerge, and to facilitate the rapport needed to examine this topic with participants over multiple interviews. Our research contributes several key insights to HIV prevention science. It challenges the static categorisation of ‘sex workers’ in favour of understanding dynamic risk trajectories. This has measurement implications, suggesting that cross-sectional studies may mischaracterise women’s HIV risk by capturing only a single moment in fluid trajectories. Our findings suggest that intervention timing may be as critical as intervention content, with specific vulnerability windows during transitions representing optimal moments for engagement. This temporal dimension has been underexplored in intervention research, which typically evaluates what works rather than when it works best. Our study highlights the need for longitudinal approaches to HIV prevention research that can capture intervention effects across women’s changing trajectories. The typical follow-up periods in most intervention studies (3–12 months) may be insufficient to capture the long-term patterns of transition documented in our life-course narratives.

Our study faced some methodological limitations that warrant consideration. The retrospective nature of our data collection introduced potential recall bias, particularly regarding the temporal sequencing of events in participants’ trajectories into sex work. However, the multiple interviews with the same participant allowed for reflection and clarification of events. The one-year follow-up period proved insufficient to fully capture the complexity and duration of transitions in sex work in real-time. Our findings suggest these transitions often unfold over extended periods, involving multiple decision points and changing circumstances that our timeframe could not completely document. A longitudinal study design spanning 3–5 years would better capture the cyclical transitions into and out of sex work. This extended timeframe would allow researchers to document changes as they occur and provide more accurate temporal data.

## Conclusions

This longitudinal life-course narrative study demonstrates how social factors influence transitions in FSW’s life trajectories, with these transitional moments often leading to increased HIV/STI risk exposure. Understanding the life-course of FSW is key to the design of interventions that can adapt to the dynamics of risks and needs of women who sell sex, ultimately contributing to more effective and targeted HIV/STI prevention and care strategies. By adopting a multifaceted and dynamically responsive approach that considers risk differentiation from a temporal as well as a spatial perspective, we can reflect the differentiated risks linked to key transitional moments as well as the socio-economic and socio-political and strengthen targeted interventions to more effectively address the multiple challenges FSW face in their daily lives.

## Data Availability

The author confirms that all data generated or analysed during this study are included in this published article.

## Footnotes

1 Rising inflation rate and currency devaluation.
